# Role of Vanadium in Cellular and Molecular Immunology: Association with Immune-Related Inflammation and Pharmacotoxicology Mechanisms

**DOI:** 10.1155/2016/4013639

**Published:** 2016-04-11

**Authors:** Olga Tsave, Savvas Petanidis, Efrosini Kioseoglou, Maria P. Yavropoulou, John G. Yovos, Doxakis Anestakis, Androniki Tsepa, Athanasios Salifoglou

**Affiliations:** ^1^Department of Chemical Engineering, Aristotle University of Thessaloniki, 54124 Thessaloniki, Greece; ^2^Laboratory of Clinical and Molecular Endocrinology, 1st Department of Internal Medicine, AHEPA University Hospital, Aristotle University of Thessaloniki, 54124 Thessaloniki, Greece; ^3^Department of Medicine, Laboratory of General Biology, Aristotle University of Thessaloniki, 54124 Thessaloniki, Greece; ^4^Department of Medicine, Laboratory of Forensic Medicine and Toxicology, Aristotle University of Thessaloniki, 54124 Thessaloniki, Greece; ^5^Forensic Medical Service of Thessaloniki, Ministry of Justice, Transparency, and Human Rights, Dimokratias 1 Square, 54012 Thessaloniki, Greece

## Abstract

Over the last decade, a diverse spectrum of vanadium compounds has arisen as anti-inflammatory therapeutic metallodrugs targeting various diseases. Recent studies have demonstrated that select well-defined vanadium species are involved in many immune-driven molecular mechanisms that regulate and influence immune responses. In addition, advances in cell immunotherapy have relied on the use of metallodrugs to create a “safe,” highly regulated, environment for optimal control of immune response. Emerging findings include optimal regulation of B/T cell signaling and expression of immune suppressive or anti-inflammatory cytokines, critical for immune cell effector functions. Furthermore, in-depth perusals have explored NF-*κ*B and Toll-like receptor signaling mechanisms in order to enhance adaptive immune responses and promote recruitment or conversion of inflammatory cells to immunodeficient tissues. Consequently, well-defined vanadium metallodrugs, poised to access and resensitize the immune microenvironment, interact with various biomolecular targets, such as B cells, T cells, interleukin markers, and transcription factors, thereby influencing and affecting immune signaling. A synthetically formulated and structure-based (bio)chemical reactivity account of vanadoforms emerges as a plausible strategy for designing drugs characterized by selectivity and specificity, with respect to the cellular molecular targets intimately linked to immune responses, thereby giving rise to a challenging field linked to the development of immune system vanadodrugs.

## 1. Introduction

**Figure 1:**
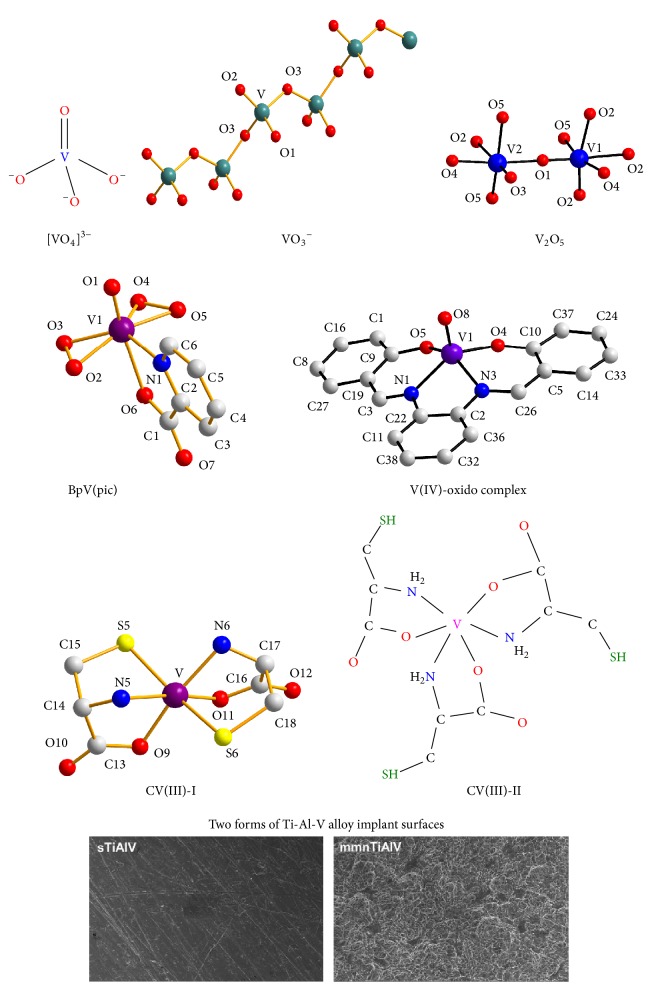
Vanadium forms exhibiting immunogenic activity.

**Figure 2:**
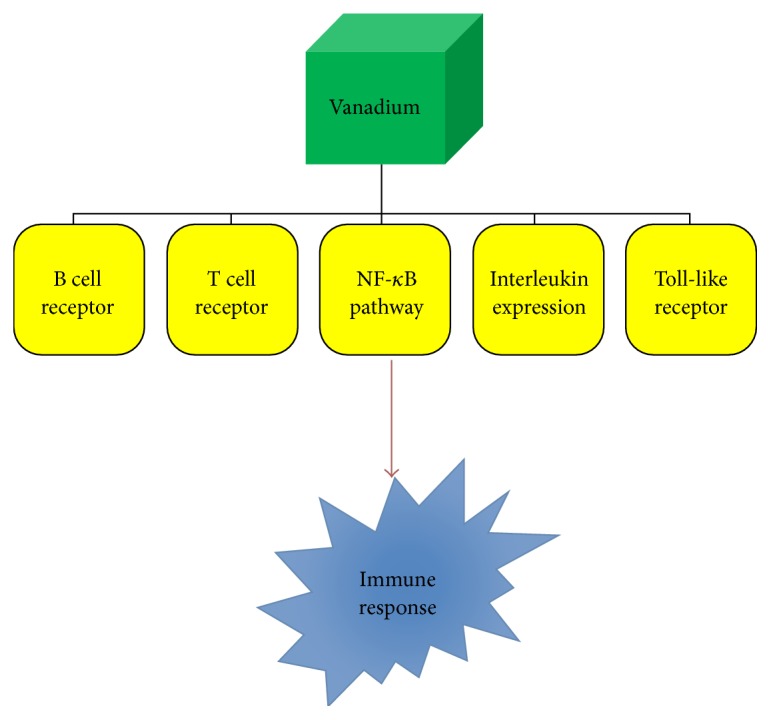
Vanadium influences several immune-related pathways, thereby sculpturing immune response.

In the past decades, several metallodrugs have been developed to target human pathophysiologies, with platinum, copper, vanadium, gold, ruthenium, and yttrium, among select metal ions, serving as the basis of such pharmaceuticals [1, 2]. Representative examples of therapeutic metallodrugs include Y-90 (Zevalin) used in the treatment of non-Hodgkin's lymphoma, sodium aurothiomalate (Myochrysine, Myocrisin, and Tauredon) or aurothioglucose (Aureotan, Solganal, Solganol, and Auromyose) used in rheumatoid arthritis patients, and bismuth subsalicylate (Pepto-Bismol), a widely used drug for the treatment of gastrointestinal disorders [1]. Beyond those, the successful platinum-based metallodrugs (cisplatin, carboplatin, and oxaliplatin) as antitumor agents were burdened by undesirable toxic side effects and appearance of chemoresistance. Both of them emerged as dire problems forcing the development of alternative metallodrugs with distinct modes of action and fewer or no side effects [3]. Vanadium is a known metal of high physiological, environmental, and industrial importance. It is an early first-row transition metal (Group 5 with *Z* = 23), with an electronic configuration of [Ar]3d^3^4s^2^, having two natural isotopes, ^51^V and ^50^V. Its presence in biological systems in the marine and terrestrial environment has been well-established over the years [4]. It is encountered, among others, in vanadium-dependent haloperoxidases and alternative nitrogenases [5]. Moreover, various vanadium species have been found to exhibit significant effects as external cofactors, inhibiting the function of a wide range of enzymes (glyceraldehyde-3-phosphate dehydrogenase, lipoprotein lipase, tyrosine phosphorylase, glucose-6-phosphate dehydrogenase, glycogen synthase, adenylate cyclase, and cytochrome oxidase) and stimulating the function of others (Na^+^-K^+^-ATPase, H^+^/K^+^-ATPase, myosin ATPase, dynein, adenylate kinase, phosphofructokinase, and choline esterase) [6, 7]. From the biological point of view, the oxidation states V(IV) and V(V) appear to be of strong interest, with cationic and anionic complexes thereof forming in the physiological pH range (pH 2–8).* In vivo*, a key redox interplay emerges between the physiologically relevant V(V) and V(IV) oxidation states, with medium equilibria defining their distribution intra- and extracellularly. V(III), on the other hand, is present in ascidians and fan worms, but it is not present in higher organisms [8, 9]. Its emergence in biological media under reduced conditions, however, leaves a lot to be scrutinized with respect to potential roles in bioprocesses [10, 11] currently elusive or unknown. Nevertheless, the majority of mammalian tissues contain approximately 20 nM vanadium. Consequently, involvement of a biogenic metal ion, such as vanadium, in immune-regulating mechanisms, including immune suppression and inflammation downregulation, formulates a well-defined platform for research into future effective and efficient immunotherapy [12, 13]. In this respect, the herein elaborated account presents new facets of the merit that vanadium holds as a metallodrug in immunotherapy, based on currently held views and knowledge emerging from ongoing research in the fields of (bio)chemical and medical interest (Figure 1). The various forms of vanadium thus far employed in immune-related pathologies (a) necessitate an orderly account of its (bio)chemical activity at the cellular and molecular level, (b) signify a structure-based elaboration of its involvement in immune system interactions and responses, and (c) point out significant factors entering future design of new vanadodrugs capable of atoxically, selectively, and specifically targeting cellular molecular loci, intimately influencing immunophysiology and contributing to immunopharmaceuticals in a host of relevant diseases (Figure 2).

## 2. The Role of Vanadium in B Cell Signaling

A number of recent studies have noted the role of vanadium in B cell signaling. This association triggers activation of multiple signaling cascades involving kinases, GTPases, and transcription factors [14]. This, in turn, results in changes in cell metabolism, gene expression, and cytoskeletal organization that regulate cellular mechanisms such as survival, tolerance, apoptosis, proliferation, and differentiation into antibody-producing cells or memory B cells. In this regard, a recent study has shown that vanadium treatment significantly proliferated splenocytes and expansion of B cells accounted for increased immune response and high number of splenocytes [15]. Vanadium treatment showed potency in amplifying the production of IFN-*γ* and total IgG in irradiated splenocytes, which correlated with the expansion of B cells. In agreement with previous reports, the number of CD3^+^, CD4^+^, and CD8^+^ cells of splenocytes was not affected. The number of CD11b^+^ and Gr-1^+^ cells in splenocytes also showed no difference upon vanadium treatment. However, the CD45R/B220^+^ B cell population expanded to significant levels in irradiated mice treated with sodium metavanadate (NaVO_3_) (Figure 1) (Table 1). Consistent with the results from irradiated mice, 0.245 *μ*M NaVO_3_ treatment markedly enlarged the population of CD45R/B220^+^ B cells of both non-irradiated and irradiated splenocytes and enhanced activation of immune B cell signaling. The effect of sodium orthovanadate (Na_3_VO_4_) (Figure 1) (Table 1) on the enhancement of DNA synthesis by T and B cell mitogenic agents was also studied using murine thymocytes and splenocytes [16]. Addition of orthovanadate to thymocyte cultures inhibited the mitogenic response in a concentration-dependent fashion. On the other hand, DNA synthesis, induced in thymocytes by pokeweed lectin and periodate treatment, was essentially uninhibited at the lower vanadate concentrations that were markedly effective for concanavalin A-induced synthesis. In addition, no significant inhibition of mitogenesis of splenic B cells in response to lipopolysaccharide and dextran was detectable at lower orthovanadate concentrations. In the absence of added mitogens, orthovanadate was found to be mitogenic for a subpopulation of thymus cells but not for splenocytes or T cell-enriched splenocyte populations. Evidently, the results suggest that (a) vanadate affects mitogenic responses in lymphocytes and (b) the interaction of vanadate with T and B cells is distinctly different, thus modulating B cell immune response. Histological studies indicating the presence of morphologically normal B cells in islets from vanadium-treated diabetic animals suggest, however, that vanadium treatment might not only mimic the effects of insulin, but also, at least partially, prevent and/or treat B cell lesions [17]. Induced vanadyl sulphate accumulation in bone, kidney, and liver prevented some alterations classically associated with diabetes, without causing further notable changes in various blood parameters or the histology of various tissues. In summary, the findings indicate that vanadium could be useful as a potential immunostimulating agent.

## 3. Regulation of T Cell Signaling

**Figure 3:**
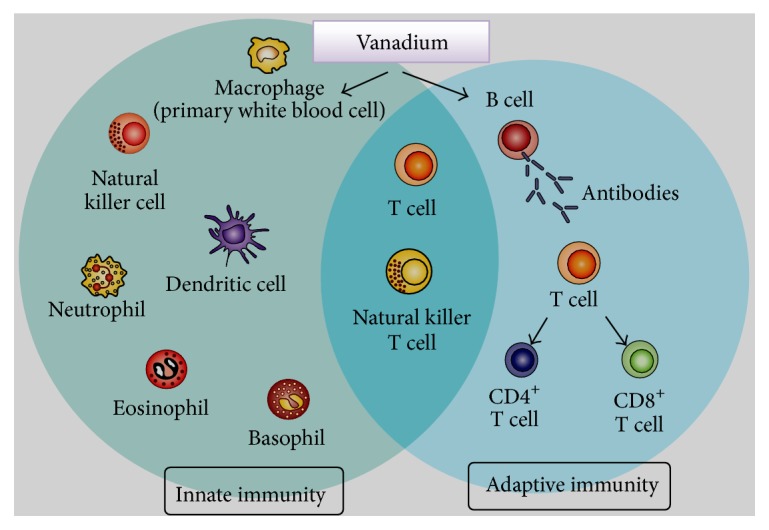
Key role(s) of vanadium in promoting innate and adaptive immunity.

Vanadium-induced immune activation also involves T lymphocytes that (a) play a central role in cell-mediated immunity and (b) are characterized by the presence of a T cell receptor (TCR) on their cell surface [18]. T cells are essential for human immunity and almost every aspect of the adaptive immune response is controlled by them. Vanadium can influence T cell signaling by changing the number of mature T cells migrating from the thymus to the spleen. Because of that, secretion of IL-2 and IL-6 is affected (Figure 3). Furthermore, ammonium metavanadate (NH_4_VO_3_) (Table 1) inhibits the proliferation activity of CD3^+^, CD3^+^CD4^+^, and CD3^+^CD8^+^ splenic T cells and depresses their activity in broilers [19]. According to this study, vanadium can affect the percentage of splenic T cell subsets, the proliferation of splenic T cells, and serum IL-2 and IL-6 content. Vanadium in excess of 30 ppm reduces T cell population, serum IL-2 and IL-6 content, and proliferation of splenic T cells, which means that cellular immune function is finally impaired in broilers. Contrary to that, vanadium concentration < 30 ppm increases the T cell population and serum IL-2 and IL-6 contents, thereby improving cellular immune function. It is speculated that vanadium influences T cell subsets by modulating the thymic selection function, as there are lesions observed in the thymus where T cells are activated and differentiated [20]. Findings reveal that vanadium can affect expression of CD3^+^, CD3^+^CD4^+^, and CD3^+^CD8^+^ T cells in both ileac lamina propria lymphocytes (LPLs) and intraepithelial lymphocytes (IELs), implying that the immune function of local intestinal mucosa in broilers could be affected by vanadium treatment. In addition, vanadium can also modify immune CD11c and MHC-II expression in thymic dendritic cells by decreasing the presence of CD11c surface marker on mouse thymic dendritic cells as a result of vanadium pentoxide (V_2_O_5_) exposure. It is surmised that this decrease might induce dysfunction, including possible negative selection of T cells, which could increase the presence of autoreactive clones in the exposed host [21]. On an equal footing, vanadium has been reported to alter CD4^+^ T helper (T_h_) cell expression, serving as an important initiator and regulator of cellular and humoral immune responses against infectious microorganisms and other antigens. Sodium orthovanadate exposure (a) enhanced inducible forms of CREB (cAMP response element-binding protein) in both resting and antigen-stimulated T cells, followed by activation of the p50/p65 heterodimeric form of NF-*κ*B, and (b) inhibited activation of NFAT (nuclear factor of activated T cells) and affected levels of its constitutive DNA-binding activity in resting lymph node T cells, whereas it enhanced AP-1 activity in transgenic mouse CD4^+^ T lymphocytes [22].

## 4. Shaping Cytokine-Interleukin Response

Cytokine interleukins belong to a family of immunomodulatory proteins that elicit a wide variety of immune responses in various tissues and organs [23, 24]. Over the past years, vanadium has been shown to interact with several IL members. A notable example is IL-2, which plays a key role in regulating immune system tolerance and immunity, primarily via its direct effects on T cells. Using an (IL-2)-independent human NK-92MI cell line that is phenotypically considered an NK bright cell line, studies have shown that vanadium pentoxide (V_2_O_5_) (Figure 1) inhibited secretion of select proinflammatory cytokines and cell proliferation, induced apoptosis, and modified the IL-2 receptor signaling pathway [25] (Table 1). Vanadium also inhibited IL-10 and IFN-*γ* secretion, but mostly only after a 24 h exposure and primarily at higher doses tested. In a similar manner, it was found that dietary vanadium in excess of 30 mg/kg (a) reduces the population and proliferation of T cells and interleukin-2 (IL-2) content in the spleen and serum and (b) causes lesions in the spleen and bursa of Fabricius in broilers [26]. Likewise, vanadium was shown to downregulate specific interleukin expression, mainly IL-6, IL-10, TNF-*α*, and IFN-*γ*, in the cecal tonsil. IL-6, as a proinflammatory cytokine, acts as a mediator of fever, acute phase response, and is responsible for stimulating acute phase protein synthesis as well as the production of neutrophils in the bone marrow. It supports B cell growth of and is antagonistic to regulatory T cells. On the other hand, IL-10 is a cytokine with numerous, pleiotropic, effects responsible for immunoregulation and inflammation. It reduces Th1 cytokine expression, MHC class II antigens, and macrophage-induced costimulatory molecules by blocking NF-*κ*B activity [23, 24]. It also promotes B cell survival, proliferation, and antibody production. Ammonium metavanadate and vanadium pentoxide (Figure 1) were shown to affect production-release of similar immunoregulatory cytokines and disrupt cell-mediated immunity. Specifically, release of the (IL-1)/(TNF-*α*)-regulating prostanoid PGE_2_ was significantly increased at the highest vanadate concentration, although LPS-stimulated PGE_2_ production was unaffected. These results indicate that,* in vitro*, pentavalent vanadium (V(V)) can interfere with immunoregulatory mediators critical for maintaining host immunocompetence [27].

## 5. Targeting the NF-***κ***B Signaling Pathway

In recent years, several studies have demonstrated that NF-*κ*B might be a very important target for vanadium with regard to the influence of cell signaling mechanisms and gene expression. Vanadium has the ability to interact with several transcription factors and influence the activity of the cell cycle, oncogenes, or tumor suppressor genes. V(IV) complex species (Figure 1) seem to promote differentiation and mineralization of the mesenchymal stem cells via activation of the NF-*κ*B/ERK signaling pathway and subsequent enhancement of the NF-*κ*B mediated action. Moreover, it has been demonstrated that ERK is implicated in the rise of the transcriptional activity of NF-*κ*B. Thus, it is possible that V(IV) modulates both ERK and NF-*κ*B pathways, and each pathway would act in concert to stimulate osteoblasts [28]. Likewise, bis(peroxido)vanadium species (Bpv) (Figure 1) (Table 1), a phosphotyrosine phosphatase inhibitor, induces myogenic cells to acquire a gene expression profile and differentiation potential consistent with the phenotype of circulating precursors, while maintaining their myogenic potential. These effects are mediated by NF-*κ*B activation through the Tyr42-I*κ*B-alpha phosphorylation, as shown by the expression of the dominant negative mutant form of the p50 NF-*κ*B subunit [29]. Moreover, treatment of macrophages with sodium metavanadate results in the activation of both NF-*κ*B and c-Jun N-terminal kinase (JNK) [30]. The activity of I*κ*B kinase-beta (IKKbeta) was significantly elevated concurrently with the increased degradation of I*κ*B-*α* and enhanced NF-*κ*B activity in cells exposed to metavanadate. Thus, both IKK and SAPK/ERK kinase 1 (SEK1), an intermediate kinase within the MEKK1 to c-Jun N-terminal kinase (JNK) cascade, are involved in vanadate-induced NF-*κ*B activation. Finally, “pervanadate” (V(V)-peroxido) was also shown to activate the DNA-binding activity of NF-*κ*B, through (a) tyrosine phosphorylation and (b) expression of the T cell tyrosine kinase p56^lck^, but not degradation of I*κ*B-*α* [31] (Table 1). Evidently, suitably configured vanadium species of both oxidation states (V(IV) and V(V)) are in a position to support distinct influence patterns of reactivity in key NF-*κ*B signaling pathways.

## 6. Subverting Toll-Like Receptor Signaling

Toll-like receptors (TLRs) constitute a distinct type of pattern recognition receptors (PRR) playing a crucial role in innate immune response [32]. Triggering TLRs to generate an immune response is therefore a primary goal in immunotherapy. To this end, certain metallodrugs are able to elicit an immune response in various immune cell types via Τoll-like receptors (TLRs) and, correspondingly, their receptor agonists [33, 34]. Recently, texture-specific vanadium-containing alloy materials (mmnTi-Al-V), reflecting implant materials, were shown to diminish TLR expression, exhibiting an 8-fold reduction in mRNAs for Τoll-like receptor-4. Treated cells had reduced levels of proinflammatory interleukins and higher mRNAs for factors strongly associated with cell apoptosis [35] (Figure 1) (Table 1). Under normal conditions, TLR ligation and dimerization activate signaling cascades and subsequent production of proinflammatory cytokines, interferons, ROS, and proteases. Signaling involves recruitment of adaptor proteins MyD88, MAL, TRIF, or TRAM. The MyD88-dependent pathway is required for all TLRs except for TLR3, and MyD88 signaling involves a serine kinase (IL-1R)-associated kinase (IRAK), TNFR-associated factor 6 (TRAF6), and (TGF-*β*)-activated kinase 1 (TAK-1) sequence followed by activation of nuclear factor NF-*κ*B and activator protein 1 (AP-1) transcription factors via the IKK and MAPK pathways, respectively [36]. TLR-targeting therapies, employing metallodrugs currently under development and clinical trials, and better understanding of the mechanisms of TLR-targeting therapies are thus expected to allow more specific treatments to be developed, thereby improving treatment options for immunoinflammatory disorders.

## 7. Role in Inflammation-Related Immunopathology

Activation of the inflammatory cascade involves immune cell mediators, transcription factors, and chemokines [37]. Inflammation is characterized by upregulation in the systemic concentrations of inflammation-related cytokines such as IL-6, IL-8, IL-18, TNF-*α*, and C-reactive protein (CRP) [38, 39]. Accumulating evidence reveals that vanadium can downregulate inflammatory reactions both* in vitro* and* in vivo*. To this end, recent findings have shown that vanadium administration reduced serum creatinine and blood urea nitrogen levels, suggesting amelioration of renal dysfunction [40]. Moreover, vanadium(III)-(L-cysteine) (VC-III) (Figure 1) (Table 1) treatment significantly prevented CDDP (*cis*-diamminedichloroplatinum(II))-induced generation of reactive oxygen species (ROS) and reactive nitrogen species (RNS) and onset of lipid peroxidation in kidney tissues of experimental mice. In addition, vanadium also substantially restored CDDP-induced depleted activities of the renal antioxidant enzymes, such as superoxide dismutase, catalase, glutathione peroxidase, glutathione-S-transferase, and glutathione (reduced) levels. Histopathological analysis also confirmed reduced expression of proinflammatory mediators such as NF-*κ*B, COX-2, and IL-6. VC-III administration also stimulated the Nrf2-mediated antioxidant defense system through promotion of downstream antioxidant enzymes, such as HO-1. Moreover, vanadium treatment significantly enhanced CDDP-mediated cytotoxicity in MCF-7 and NCI-H520 human cancer cell lines. Thus, VC-III can serve as a suitable chemoprotectant and increase the therapeutic window of CDDP in cancer patients. Furthermore, bis(peroxido)vanadium is able to prevent neuronic inflammation on cerebral ischemia. Data reveal that bis(peroxido)vanadium (Bpv), a specific inhibitor of PTEN's phosphatase activity, exhibits powerful neuroprotective properties [41]. Treatment with Bpv significantly increased IL-10 levels and decreased TNF-*α* concentration in the ischemic boundary zone of the cerebral cortex. Likewise, vanadium(III)-(L-cysteine) treatment significantly reduced PTEN mRNA and protein levels and increased PI3K, Akt, and p-GSK-3*β* protein expression in the ischemic boundary zone of the cerebral cortex. These results (a) demonstrate the neuroprotective effects of bis(peroxido)vanadium on cerebral ischemia and reperfusion injury of ischemic stroke rats and (b) show that vanadium is associated with reduction of inflammatory mediator production and upregulation of PTEN downstream proteins PI3K, Akt, and p-GSK-3*β*.

## 8. Pharmacotoxicology Mechanisms

**Figure 4:**
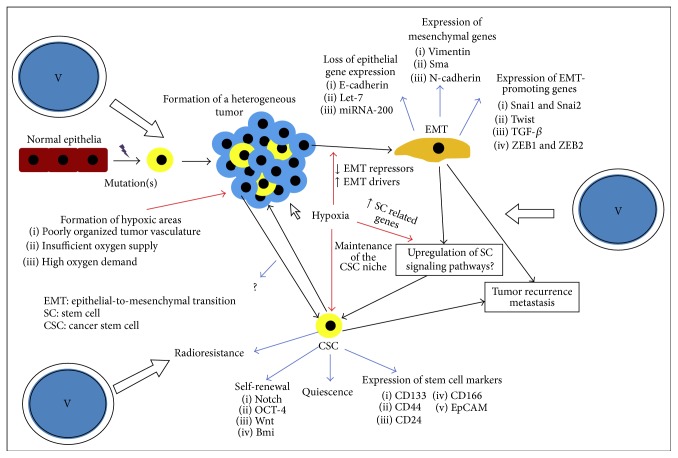
Current obstacles to overcome by specifically designed vanadium metallodrugs in cancer immunotherapeutics.

Increasing evidence shows that complex vanadium species possess structural characteristics that justify their chemical reactivity at the biological level, thereby rendering them viable candidates for immune system disease metallodrugs [42, 43]. In order for vanadium compounds to be effective, atoxic well-defined forms of that metal ion encompassing selected physicochemical characteristics should be examined carefully in terms of their availability, selectivity, and specificity, followed by long-term epidemiological studies and controlled clinical trials. For such well-defined forms to emerge as immunomodulatory agents, key factors should be taken into consideration in the design and subsequent synthetic efforts. Such factors include (a) the nature of vanadium itself (inorganic forms at various oxidation states, metal-organic complex species, organometallic forms, etc.), (b) the nature of ligands-substrates bound to vanadium (e.g., peroxido, oxido, and nonperoxido organic chelators of variable O,N-containing tethers), (c) the oxidation state of vanadium (with V(IV) and V(V) representing the well-established physiological forms in human biological fluids, and V(III) awaiting further perusal), (d) the hydrophilicity-hydrophobicity of the ligands-substrates as well as the arising vanadium complex inorganic-organic species, thereby allowing access to specified molecular loci of action, and (e) the binary and ternary complex metal-organic nature of vanadium bestowing appropriate chemical reactivity where and when such is needed to counteract carcinogenic activity. The aforementioned collective properties formulate the chemical profile of vanadium that will configure its biological reactivity and consequently adhere to the selectivity and specificity needs of the immune system target site(s) of anti-inflammatory action. The need for such approaches to new atoxic vanadium compounds exemplifies the motivation for commensurate research efforts currently underway (Figure 4). In line with the emergence of select vanadium species, capable of delivering immunogenic activity, studies on the identification of immune system specific sites of interaction of vanadium with biomolecular targets in the cell should be conducted, shedding light onto the chemistry associated with the biological activity of vanadium in its various selected atoxic forms (Table 1). Current research data presented in this review highlight vanadium's synthetic and structural bioinorganic profile along with its biological activity attributes, collectively formulating the significant potential of unique structure-based and immune process-specific vanadodrugs for the detection, prevention, and treatment of immune system aberrations.

## 9. Conclusions

Overall, specified vanadium complex species are involved in key mechanisms of immune regulation and can be used as promising metallodrugs toward future immunotherapy. Therefore, significant merit emerges toward further studies attempting to (a) design new vanadodrugs and (b) decipher the potential role that vanadium species have in interactions with immune system modulators as well as other transcription factors influencing immune signaling. Concurrently, vanadium regulation of B and T cell signaling emerges as a useful tool in probing modulatory mechanisms of inflammation suppression and their (in)direct implication in immunotherapeutic approaches. In addition, activation of certain interleukins, including IL-2, IL-4, IL-6, and IL-10 by vanadium denotes their specific contribution to immunometabolic processes, thereby warranting further perusal into the development of diagnostic and immunotherapeutic tools in immunopathological disorders. Numerous advances have contributed to the understanding of the cellular and molecular pathways involved in immune-related inflammation and stand as groundwork toward further investigations linking interleukin involvement to inflammation-driven immune response. Sculpting the immune response using metallodrugs may thus be a challenging goal toward future immunotherapies. The collective data mentioned in the current review reflect apt examples of vanadium-based approaches in cancer immunotherapy and related diseases. To this end, better understanding of the molecular signaling pathways used by vanadium interjection in immune surveillance, immune-driven inflammation, and immune cells stands as a well-defined platform for targeted research into future effective and efficient vanadium-based immunotherapy. Defined into such a well-formulated framework, vanadium-linked approaches in immunotherapy have merit, deserve due attention, and warrant further investigation.

## Figures and Tables

**Table 1:** Vanadium oxidation state (V(V, IV, III)) and form-specific effects in immune system processes (^A^ *in vitro*, ^B^ *in vivo*).

Vanadium species, compound	Immune system process, pathology, disease	Vanadium effect	Vanadium dose
Sodium metavanadate (V(V))	Immune system activation against *γ*-irradiation in mice	(1) Amplification of production of IFN-c and total IgG in irradiated splenocytes (2) Expansion of B cells accounting for increased number of splenocytes	0–3.99 *μ*M^15,B^
	NF-*κ*Β/JNK signaling	Activation of both NF-*κ*B and c-Jun N-terminal kinase (JNK)	0–80 *μ*M^30,A^

Ammonium metavanadate (V(V))	T cell signaling	(1) Concentration-dependent inhibition-proliferation of splenic T cells	5–60 ppm^19,B^
		(2) Immune system function of local intestinal mucosa in broilers could be affected	5–60 mg/kg^20,B^
	Cellular immune function	Reduction of percentage of peripheral blood T-cell subsets and proliferation function and serum interleukin-2 content	5–60 ppm^26,B^

Vanadium pentoxide (V(V))	T lymphocyte activation	(1) Inhibition of secretion of proinflammatory cytokines (IL-1, TNF-*α*, etc.)	1 fM–100 *μ*M^27,A^
Ammonium metavanadate (V(V))	Immunocompetence	(2) Effect of production-release of major immunoregulatory cytokines and disruption of cell-mediated immunity	

Vanadium pentoxide (V(V))	Autoimmunity	(1) Thymic dysfunction (2) T cell negative selection in mice	0.02 M^21,B^
	Impairment of function of immunoregulatory NK cells	IL-2-mediated dysregulation of signaling pathways in NK cells	25–400 *μ*M^25,Α^

Sodium orthovanadate (V(V))	B cell signaling	Enhancement of DNA synthesis by T and B cell mitogenic agents	0–1000 *μ*M^16,B^
	T cell signaling	(1) Enhancement of inducible forms of CREB in both resting and antigen-stimulated T cells (2) Enhancement of AP-1 activity in primary T lymphocytes	10–100 *μ*M^22,Β^

“Pervanadate” (V(V)-peroxido species)	NF-*κ*Β signaling	NF-*κ*B activation through tyrosine phosphorylation	50–250 *μ*M^31,Α^

Bis(peroxido)vanadium species (Bpv) (V(V))	NF-*κ*Β signaling	(1) NF-*κ*B activation	10 *μ*M^29,Α^
		(2) Neuroprotection	0.2 mg/kg/day^41,B^

Vanadyl sulphate (V(IV))	B cell morphology	(1) B cell morphology maintenance (2) Prevention and/or treatment of B cell lesions induced by streptozotocin treatment	0.25, 0.50, 0.75, 1.00 mg/mL^17,B^

Vanadium(IV) oxido complex N,N′-Bis(salicylidene)-O-phenylenediamine vanadium(IV) oxide (V(IV))	NF-*κ*Β signaling	Modulation of both ERK and NF-*κ*B pathways	7–25 *μ*M^28,Α^

Vanadium(III)-(L-cysteine) (V(III))	Antioxidant defense, inflammation	(1) Prevention of cisplatin generation of ROS (2) Restoration of renal antioxidant enzymes (3) Chemoprotectant in cisplatin therapy	1–10 *μ*M^40,Α^ 1 mg/kg^40,B^

Ti-Al-V alloy surfaces	TLR signaling	(1) Reduction of TLR4 mRNA (2) Proinflammatory interleukins, cell death, and apoptosis	N/A^35,Α^

## Abbreviations

ATPase: Adenosine triphosphatase
GTPase: Guanosine triphosphatase
AP-1: Activator protein 1
LPLs: Lamina propria lymphocytes
IELs: Intraepithelial lymphocytes
IFN-*γ*: Interferon-gamma
TCR: T cell receptor
TNF-*α*: Tumor necrosis factor alpha
MHC: Major histocompatibility complex
PGE_2_: Prostaglandin E_2_
ERK: Extracellular signal-regulated kinases
IKKB: Inhibitor of nuclear factor kappa-B kinase subunit beta
JNK: c-Jun N-terminal kinase
MEKK1: Mitogen-activated protein kinase kinase kinase 1
SAPK: Stress-activated protein kinase
NF-*κ*B: Nuclear factor of kappa light polypeptide gene enhancer in B cells
NFAT: Nuclear factor of activated T cells
CDDP: * cis*-Diamminedichloroplatinum(II)
MYD-88: Myeloid differentiation primary response gene 88
COX-2: Cyclooxygenase-2
Nrf-2: Nuclear factor (erythroid-derived 2)-like 2
CRP: C-reactive protein
Bpv: Bis(peroxido)vanadium
PTEN: Phosphatase and tensin homolog
PI3K: Phosphoinositide 3-kinase
Akt: Serine-threonine kinase-protein kinase B
p-GSK-3*β*: Phosphorylated glycogen synthase kinase 3 beta
mmnTi-Al-V: Macro-/micro-/nanotextured rough Ti-Al-V.
